# Editorial: Role of glia in neurodevelopmental disorders

**DOI:** 10.3389/fncel.2024.1404362

**Published:** 2024-04-11

**Authors:** Marine Krzisch, Simona D'Antoni, Lucia Ciranna

**Affiliations:** ^1^School of Biomedical Sciences, Faculty of Biological Sciences, University of Leeds, Leeds, United Kingdom; ^2^Institute for Biomedical Research and Innovation, National Research Council (IRIB-CNR), Catania, Sicily, Italy; ^3^Department of Biomedical and Biotechnological Sciences, Faculty of Medicine, University of Catania, Catania, Sicily, Italy

**Keywords:** glial cells, neurodevelopmental disorders, microglia, astrocytes, neurological disease

This Research Topic highlights recent advancements in our understanding of the role of glia in neurological disease, with a special focus on neurodevelopmental disorders. This is an emerging field in neuroscience, as glial cells were long considered to have a simple support role in the brain. The Research Topic is composed of two research articles and two reviews, highlighting the role of glial cells in different neurodevelopmental disorders.

Tuberous Sclerosis Complex (TSC) is a genetic disorder characterized by the loss of function of either TSC1 or TSC2 genes, resulting in the hyperactivation of the mechanistic target of rapamycin (mTOR) pathway at the molecular level. mTOR dysregulation in the brain leads to altered cortical development, resulting in the formation of focal lesions known as tubers, which are associated with a wide range of neurological manifestations, including epilepsy. Little is known about how astrocytes contribute to epilepsy in this disease.

The study by Luinenburg et al. investigates the disease-relevant phenotypes of astrocytes differentiated from TSC patient-derived induced pluripotent stem cells and cultured in two dimensions. TSC astrocytes showed reduced maturity and were unable to clear excess extracellular glutamate, through decreased expression of glutamate transporters and receptors, glutamine ligase, and adapter protein as well as a reduction in their phagocytic activity. This research furthers our understanding of the defects of astrocytes in TSC, and provides new potential avenues for TSC treatment, focused on astrocytes.

The review by Talvio and Castrén focuses on the dysfunction of astrocytes in Fragile X syndrome (FXS), a neurodevelopmental disorder caused by the lack of Fragile X Mental Retardation Protein (FMRP). It is one of the leading causes of intellectual disability and autism. Similarly to the Luinenburg et al. study, this review points to altered dynamics of astrocytic maturation in FXS. It provides evidence for other cell-autonomous astrocytic phenotypes involved in the pathogenesis of FXS, such as altered proliferation, calcium signaling, lipid homeostasis and inflammatory activity. Furthermore, FXS astrocytes affect synaptic function and may be involved in abnormal neuronal development in FXS.

Autism spectrum disorder (ASD) is a complex neurodevelopmental disorder and growing evidence suggests that glial cell dysfunction may contribute to its pathophysiology. Furthermore, energy metabolism is crucial in normal brain development and metabolic alterations contribute to different neurodevelopmental disorders. The review by Cantando et al. describes the metabolism of astrocytes and microglia in the developing postnatal brain and its importance in regulating the maturation of both glial cells and neuronal circuits in the brain. Authors also discuss the possible involvement of these cells in the metabolic abnormalities observed in ASD brains. Dysregulation of metabolic pathways in glial cells, such as mitochondrial dysfunction, alterations in lipid metabolism and glycolytic shifts, can affect neuronal function and can contribute to the neurodevelopmental and behavioral manifestations related to ASD.

The article by Rodríguez-Pérez et al. focuses on the mechanisms underlying the coexistence of congenital hydrocephalus and corpus callosum dysgenesis in many neurodevelopmental syndromes. Corpus callosum dysgenesis was previously believed to be a consequence of ventricular dilation. Interestingly, using a mouse model for congenital hydrocephalus (hyh mutant mouse), the authors instead showed that corpus callosum dysgenesis appears before hydrocephalus and identified a specific and significant malfunction of the glial wedge, a midline cell population deputed to provide guidance cues for developing axons. The development of neurons generating callosal axons was not disrupted in the hyh mutant mouse; however, when pioneering axons approached the area corresponding to the deficient glial wedge population, they turned toward the ipsilateral lateral ventricle, failing to cross the interhemispheric midline like in wild-type mice. In addition, the authors suggest that the radial glial cell defect also causes hydrocephalus in the hyh mutant mouse, opening a new hypothesis that should be investigated in human pathology.

Altogether, this Research Topic highlights data from different models of glial cells and different neurodevelopmental disorders to offer new perspectives on the role of glial cells in these diseases. Because the interest in glial cells related to neurological disease is new, we expect that many more advances will be achieved in the future. Targeting glial cells could provide novel therapeutic avenues for intervention in neurodevelopmental disorders. We hope that this Research Topic will inspire scientists working in this field to address the missing knowledge on the role of glial cells in neurodevelopmental disorders, and further our understanding of the dysfunction of these cells and their impact on other cell types in the brain.

## Author contributions

MK: Writing – original draft, Writing – review & editing. SD'A: Writing – review & editing. LC: Writing – review & editing.

